# Letter to the editor regarding the article ‘kidney tissue elastography and interstitial fibrosis observed in kidney biopsy’

**DOI:** 10.1080/0886022X.2022.2048018

**Published:** 2022-03-07

**Authors:** Ziman Chen, Jiaxin Chen, Zhongzhen Su

**Affiliations:** Department of Ultrasound, Fifth Affiliated Hospital of Sun Yat-Sen University, Zhuhai, China

Dear Editor,

We read with great interest the recently published article by Islamoglu MS et al. [1] entitled ‘Kidney tissue elastography and interstitial fibrosis observed in kidney biopsy’. The study aimed to investigate the relationship between tissue stiffness detected in shear wave elastography (SWE) and interstitial fibrosis observed in kidney biopsy. The authors propose a significant relationship between the SWE measurements and interstitial fibrosis. Although we appreciate the authors for their hard work on the construction of a study to enhance our understanding of the possible association of renal fibrosis with renal stiffness derived from SWE, some methodological notes should be pointed out to make this study more reasonable and practical, at least theoretically.

First, the authors pointed out that the SWE procedure was performed using a sonoelastography device (Aixplorer; SuperSonic Imagine, Aix-en-Provence, France). However, to our knowledge, the images (Figures 1 and 2) [1] presented in this study were not derived from Aix-en-Provence Ultrasound system [2]. The authors need to clarify the elastography system used as the accuracy of the results could be affected by different elastic imaging systems [3]. Second, the SWE values were obtained from the inferior pole of the kidney in this study. However, due to the nature of renal anisotropy, the difference of elastic measurements between renal poles and mid-region was significant, which was related to the angle between the ultrasound beam and the cortex [4]. The success rate of measurements in the renal poles was low compared with measurements in the mid-portion of the renal parenchyma and suggested that the renal poles should be avoided during measurements to ensure good reproducibility [5]. Third, in this study, no effective measures were taken to control confounding factors during SWE procedure. Patients need to be instructed to hold their breath in order to minimize motion artifacts when taking elastic measurements. Additionally, patients were requested to empty their bladder prior to SWE examination to avoid the influence induced from the filling renal pelvis.

Due to the lack of rigorous methodology, the conclusion of this study may not be reliable in fact. Recently, a study conducted by Chen Z et al. had demonstrated that the SWE derived elastic values reduced as pathology grade of renal fibrosis or grade of vessel wall thickening progresses in patients with CKD, which may be attributed to renal hypo-perfusion rather than tubulo-interstitial fibrosis progression [2]. Another study performed by Güven AT et al. also shown that magnetic resonance elastography-derived stiffness values were lower in patients with chronic injury [6]. The authors need to further explain why their findings were inconsistent with the above studies.

It's because of the nature of renal anisotropy which may affect the application of SWE in the evaluation of renal fibrosis, a more scientific and rigorous experimental protocol needs to be conducted to unveil the true relationship between SWE-derived stiffness and renal fibrosis.
